# University students’ and staff attitudes toward the implementation of a “tobacco-free” policy: A view from Qatar

**DOI:** 10.1016/j.pmedr.2024.102605

**Published:** 2024-01-09

**Authors:** Ghadir Fakhri Al-Jayyousi, Mujahed Shraim, Diana Alsayed Hassan, Mohammed Al-Hamdani, Rana Kurdi, Noor Ahmed Hamad, Hanan F. Abdul Rahim

**Affiliations:** Department of Public Health, College of Health Sciences, QU Health, Qatar University, P.O. Box 2713, Doha, Qatar

**Keywords:** Tobacco-free, Health policy, Health-promoting campus, Youth, Attitudes, Eastern Mediterranean Region

## Abstract

**Objectives:**

Tobacco-free campus policies are needed to create environments conducive to prevention or quitting in the Eastern Mediterranean Region (EMR), where the use of various tobacco products is on the rise among university students. The objectives of this study were to assess overall support for a 100% tobacco-free campus policy and its predictors among different stakeholder groups at the largest national university in Qatar and to understand attitudes towards specific policy components.

**Study design:**

A cross-sectional online survey of faculty and students.

**Methods:**

We measured sociodemographic variables, tobacco use, and support for components of a 100% tobacco-free campus policy. A total score for support was calculated, and associations with selected predictors were assessed using multivariable linear regression.

**Results:**

413 respondents participated in the survey. A majority of faculty/staff and students (76.6% and 75.5%, respectively) supported the implementation of a 100% tobacco-free campus policy, with a majority supporting extension of the policy beyond cigarettes. Support for other components of the policy varied. Among students, overall support was lower among males and tobacco users and higher among the married. Among faculty and staff, support was lower among Qatari nationals and tobacco users.

**Conclusion:**

In the context of a growing tobacco crisis in the EMR, implementing and understanding the impact of tobacco-free campus policies is essential. This paper underscores the importance of addressing diverse stakeholder attitudes and providing empirical evidence to guide policy implementation and evaluation.

## 1 Introduction

Tobacco use is one of the world's deadliest public health hazards killing approximately 8 million people annually, with around seven million deaths traced back to tobacco use and 1.2 million deaths due to inhaling secondhand smoke (Tobacco, 2021). Worldwide, cigarette smoking is the most popular method of tobacco use. Other common tobacco products include waterpipe tobacco, various smokeless tobacco products, cigars, pipe tobacco, and bidis (a form of cut tobacco rolled in leaf) (Tobacco, 2021).

According to the World Health Organization's global report on tobacco smoking trends 2000–2025, one-fifth (19.9 %) of youth aged 15 and older (33.7 % males and 6.2 % females) smoke tobacco (WHO, 2018). In the Eastern Mediterranean Region (EMR), one of the world's fastest-growing regions in terms of tobacco-product users, the prevalence of tobacco use among adults (≥15years) was 18.1 % (34.0 % males and 2.2 % females) in 2016 (WHO, 2018). This could be due to the rise in popularity of tobacco products like waterpipe (shisha) (Baroud et al., 2021) and midwakh (Al-Houqani et al., 2012, Jawad et al., 2019), a special tobacco blend (called *dokha*) smoked in an elongated pipe (Vupputuri et al., 2016). In Qatar, a country of approximately 2.7 million people in the Arab Gulf (Qatar: Planning and Statistics Authority, 2023), the prevalence of tobacco use among adults was 12.6 % (Global Adults Tobacco Survey, 2013).

Middle Eastern countries face a serious public health issue with tobacco usage among university students. A recent *meta*-analysis across universities in Saudi Arabia found that 17 % of participants were tobacco smokers (Alotaibi et al., 2019). In the United Arab Emirates, the prevalence of smoker students was 15.1 %, with roughly 44.0 % of those using e-cigarettes and 47.5 % smoking “midwakh” (Ahmed et al., 2021). A recent study among Qatar University (QU) students found that 25.6 % of students reported being current smokers, with 70.6 % using waterpipes and 54.9 % using e-cigarettes (Al-Jayyousi et al., 2021).

### 1.1 Policies at universities

Campus-wide tobacco control programs may include measures for a smoke-free campus (prohibition of smoking in all indoor and outdoor places) or tobacco-free campus (prohibition on smoking as well as on the use of smokeless tobacco products in all indoor and outdoor spaces), which may also include a prohibition on all tobacco marketing, sponsorship, and sale activities (Wang et al., 2018). Smoke-free policies implemented in universities are associated with lower rates of tobacco use by college students (Seo et al., 2011) as well as less littering of used cigarettes (Lee et al., 2013). Furthermore, stronger tobacco-free policies are linked to fewer students reporting intention to smoke on campus and fewer students reporting exposure to secondhand smoke (Fallin et al., 2015). In 2017, the United States had 2,082 and 1,743 campuses with smoke-free or tobacco-free regulations, respectively (Wang et al., 2018). Worldwide smoke-free regulations have been implemented at a number of universities, including those in New Zealand, Australia, and the United Kingdom (Robertson and Marsh, 2015, Lupton and Townsend, 2015, Burns et al., 2013). However, these regulations appear to exist on only a small number of campuses in Middle Eastern countries. The American University of Beirut (AUB) in Lebanon became the first Lebanese university to implement a full tobacco-free policy in 2018 (Farran et al., 2021), and following government guidelines King Saud University, one of Saudi Arabia's major institutions of higher education, also established a smoke-free campus policy (Almutairi, 2014).

To understand the impact of tobacco-free policies, a growing number of studies have evaluated attitudes and support towards their establishment on university campuses. Nearly 70 % of staff and students in the UK favored a tobacco-free campus prior to the establishment of a tobacco policy (Bartington et al., 2020). A surprising finding in the same study was that less than half of those polled supported a ban on e-cigarettes (Bartington et al., 2020). A *meta*-analysis of 19 studies found that 58.9 % of students and 68.4 % of faculty from the US and UK supported smoke-free policies (Lupton and Townsend, 2015). Recent research from AUB shows positive changes in staff and faculty attitudes, and perceptions of policy benefits after one year of the implementation of a tobacco-free policy in the university (Farran et al., 2021). In Qatar, a recent study from Qatar University, the country’s largest institution of higher education, indicated that 77.2 % of students supported a tobacco-free campus, but support was much lower among tobacco users compared to non-users (35.9 % and 91.8 %, respectively) (Al-Jayyousi et al., 2021).

Support for tobacco-free campus policies is not universal and is influenced by a number of factors, including socioeconomic levels, peer influence (Zaleski and Aloise-Young, 2013), and smoking status (Bartington et al., 2020, Forden and Carrillo, 2016). Males are less supportive of tobacco-free policies than females, and older age is associated with more favorable attitudes toward tobacco-free policies (Braverman et al., 2015, Cooper et al., 2016, Ickes et al., 2019, Borland et al., 2006). Mamudu et al. (2012) found that faculty members with higher levels of education and income are more likely to support tobacco control policies at universities (Mamudu et al., 2012).

### 1.2 Tobacco policies in Qatar

The State of Qatar ratified the Framework Convention on Tobacco Control, an evidence-based treaty that underlines everyone's right to the best possible quality of health, in 2004 (WHO report on the global tobacco epidemic, 2013). Qatar's anti-tobacco law, Act no. 20 of 2002, prohibited tobacco advertising in all media, as well as the import or use of cigarette vending machines. The law also prohibited smoking in enclosed public spaces, such as schools, hospitals, government institutions, and restaurants, the sale of tobacco products within 500 m of educational institutions, and the sale of cigarettes to children below the age of 18. In 2016, Act no. 20 was repealed by Law no. 10, which included broader bans on advertisements and promotional activities for cigarettes or tobacco and its derivatives (Ministry of Public Health in Qatar, 2016).

Qatar University campus is home to around 23,000 students (67 % Qatari nationals, 75 % female, and 90 % at the undergraduate level) and approximately 3000 employees, of whom 56 % are academic employees.^1^ In 2013, Qatar University enacted a no-smoking policy covering faculty, students, workers, and campus visitors. The policy prohibits tobacco smoking inside or outside buildings or in vehicles, and 'No Smoking' signs have been placed at the entrances of all buildings. Smoking is permitted in specific places that are at least 7.6 m away from QU buildings and are equipped with adequate cigarette receptacles. In 2019, QU became a part of the fifth cohort of the Tobacco Free Generation Campus Initiative, a program of the American Cancer Society's Tobacco Control Center that provides funding to “accelerate and expand the adoption and implementation of 100 percent smoke- and tobacco-free policies on college and university campuses across the nation.” It was the first and only foreign institution to join this cohort, with the goal of transitioning to a completely smoke-free and tobacco-free campus.

The attitudes of QU students towards the implementation of a comprehensive tobacco-free policy have been explored in a previous study, although the response rate to that survey was low (Al-Jayyousi et al., 2021). The current study expands on the previous one by including a larger sample size of students, examining staff member’s attitudes towards the tobacco-free policy (in addition to students), including variation by type of tobacco product, and measuring support both using a single question as well as a more detailed battery of statements regarding the policy components.

## 2 Methods

### 2.1 Study design, setting, and population

We conducted a cross-sectional study among QU community (staff, faculty, and students) using an online survey in the spring semester of 2022. All members of QU community were invited to participate in the study by email detailing the purpose of the study and a link to study information sheet and the questionnaire. The Institutional Review Board of QU approved the study (QU-IRB 1620-E/21).

### 2.2 Data collection

Data were collected anonymously using an online self-administered questionnaire in either English or Arabic, based on the respondent’s preference. The questionnaire was adapted from the Global Adult Tobacco Survey Qatar 2013, American Cancer Society Tobacco-Free Generation Campus Initiative: Cohort 5 Student Survey (2020–2021), and QU’s no-smoking policy. Bilingual research team members translated the questions into Arabic. To evaluate item clarity and effectiveness, both versions were pretested on a group of students.

### 2.3 Variables

Most variables were collected for both students and faculty/staff, while a few questions were specific to each category of respondents. We collected the following sociodemographic variables from both categories of respondents: age, sex (female/male), level of education (undergraduate/graduate below PhD/PhD), marital status (single/married/divorced or separated), nationality (Qatari/other nationality), and monthly household income (<10,000, 10,000–19,999, 20,000 – 30,000, >30,000 Qatari Riyal (QAR). Age was categorized into four age groups (based on quartile values) for students (18–19, 20–21, 22–24, and >24 years) and staff (<34, 34–40, 41–48, and >48 years).

Tobacco-related questions included current use of tobacco products (yes/no), type of tobacco product/s used (traditional cigarettes/electronic cigarettes/heat stick products/chewable tobacco/Medwakh/waterpipe (shisha)/nicotine pouches/other), current smoking status (yes/no) for family members (mother/father/sibling/spouse) and close friends. Students were asked about their college and about living arrangements (living with parents/not living with parents). Staff were asked about their job type (faculty/administrator/staff) and living arrangements (living in own household/living with parents).

Attitude scores were assessed using fourteen statements detailing the proposed components of the policy, measured on a 5-point Likert scale (strongly support = 4, support = 3, neutral = 2, do not support = 1, strongly do not support = 0). The fourteen statements included three negatively worded statements, and therefore the scoring for the three negatively worded statements was reversed. The total score ranged between 0 and 56, with higher scores indicating more supportive attitudes toward implementation of a tobacco-free policy. The same 5-point Likert scale was used to measure participant’s overall attitudes towards support for a 100 % tobacco-free and smoke-free campus using a single question “*To what extent do you support your campus becoming 100 % smoke-free, tobacco-free, and vape-free, with all tobacco product use prohibited on campus*?”.

### 2.4 Statistical analysis

Tobacco products were re-categorized into five groups (traditional cigarettes only/electronic cigarettes only/traditional and electronic cigarettes/other tobacco products/none), as some types of products were used infrequently.

Data were summarized using frequencies and percentages. Linear regression (both univariable and multivariable) analyses were conducted to test the associations between predictor variables and the total attitude score. Associations with p ≤ 0.05 were considered statistically significant. Data were analyzed using STATA. V.17.

## 3 Results

In total, 413 respondents participated in the survey, including 306 students and 107 faculty/staff (Table 1). The majority of students were between 18 and 25 years (83.6 %), female (62.4 %), studying at the undergraduate level (89.9 %), and single (87.6 %). Over one half (57.5 %) of student respondents were non-Qatari, living with their parents (91.5 %), and reporting a monthly household income of more than 8219 USD (35.6 %). About a quarter of students (23.9 %) reported having a sibling who smokes, and 24.5 % reported that they were using tobacco products at the time of the survey, mostly traditional cigarettes (18.0 %). Most of the faculty/staff respondents were above 25 years of age, female (58.9 %), faculty members (52.3 %), holding Master or PhD degrees (81.4 %), and married (75.7 %). In addition, most were non-Qatari (78.5 %), earning more than 8219 USD (43.9 %), and living in their own households (61.7 %). About a third of faculty/staff reported having a sibling who smokes and about a quarter (25.2 %) reported using tobacco products. As with students, traditional cigarettes (15.9 %) were the most common type of tobacco product used by faculty/staff, while none reported the use of Medwakh (compared to 8.8 % of students).

**Table 1:** Socio-demographic characteristics and tobacco use among participants at Qatar University, 2022 (n = 413).

	Students (n = 306) N (%)	Staff (n = 107) N (%)
Age (years) – students		
18–19	87 (28.4)	
20–21	96 (31.4)	
22–24	61 (19.9)	
>24	62 (20.3)	
Age (years) – staff		
<34		30 (28.0)
34–40		26 (24.3)
41–48		28 (26.2)
>48		23 (21.5)
Gender		
Female	191 (62.4)	62 (58.9)
Male	115 (37.6)	44 (41.1)
Education level		
Undergraduate	275 (89.9)	20 (18.7)
Graduate below PhD	22 (7.2)	45 (42.1)
PhD	9 (2.9)	42 (39.3)
Nationality		
Non-Qatari	176 (57.5)	84 (78.5)
Qatari	130 (42.5)	23 (21.5)
Marital status		
Single	268 (87.6)	21 (19.6)
Married	37 (12.1)	81 (75.7)
Separated/divorced	1 (0.3)	5 (4.7)
Monthly household income (QAR)^a^		
<10,000	61 (19.9)	4 (3.7)
10,000–19,999	90 (29.4)	27 (25.2)
20,000–30,000	46 (15.0)	29 (27.1)
>30,000	109 (35.6)	47 (43.9
College – students		
Art and Science	66 (21.6)	
Business and Economics	39 (12.7)	
Health Colleges	64 (20.9)	
Law, Education, Sharia and Islamic Studies^b^	57 (18.6)	
Engineering	80 (26.1)	
Current job – staff		
Administrator		15 (14.0)
Faculty		56 (52.3)
Staff		32 (29.9)
Other		4 (3.7)
Currently using any tobacco products		
No	231 (75.5)	80 (74.8)
Yes	75 (24.5)	27 (25.2)
At least one family member is a smoker		
No	191 (62.4)	56 (52.3)
Yes	115 (37.6)	51 (47.7)
At least one close friend is a smoker		
No	178 (58.2)	60 (56.1)
Yes	128 (41.8)	47 (43.9)
Place of living – students	280 (91.5)	
With parents	26 (8.5)	
Without parents		
Place of living – staff		
I have my own household		66 (61.7)
I live with my parents		41 (38.3)
Currently used tobacco products^c^		
Traditional cigarettes	55 (18.0)	17 (15.9)
Electronic cigarettes	25 (8.2)	7 (6.5)
HeatStick	8 (2.6)	0 (0.0)
Chewable tobacco	2 (0.7)	0 (0.0)
Medwakh	27 (8.8)	0 (0.0)
Waterpipe/shisha	33 (10.8)	13 (12.1)
Nicotine pouches	19 (6.2)	2 (1.9)
Others	3 (1.0)	0 (0.0)

a QAR = Qatari Riyal; 1 USD = 3.64 QAR.

b Colleges with less than 30 respondents were combined.

c Response frequencies out of the respondents who reported using any tobacco product; multiple answers allowed.

Two-thirds (67.4 %) of students and three-quarters of faculty/staff (74.8 %) agreed (support or strongly support) with the statement that “No one, including faculty, staff, students, visitors, and contractors, should be allowed to smoke anywhere on campus” (Table 2). When detailing where smoking should be allowed on campus, approximately one third of faculty/staff (37.4 %) and of students (31.1 %) supported allowing smoking outdoors. For indoor smoking, 78.5 % of faculty/staff and 68.6 % of students supported not having indoor designated smoking areas at QU. Around three-quarters of students and faculty and staff agreed that the policy should include any tobacco product such as e-cigarettes and chewed tobacco. The majority of students (81.0 %) and faculty/staff (86.0 %) agreed that the policy should be broadcast to the entire QU community. Around 72 % of students and 58.9 % of faculty/staff supported having the policy incorporated in faculty and staff contracts. Most participants also agreed on having the policy presented during student orientation and included in student handbooks, in contractor contracts, and in induction programs for new contractor employees. It is also worth noting that 70.3 % of students and 77.6 % of faculty/staff supported having penalties for persons violating the tobacco policy. Finally, around three-quarter of participants thought that penalties should be introduced gradually into the policy implementation to assist with compliance.

**Table 2:** Attitudes toward the implementation of a tobacco-free policy and support for a 100% tobacco-free and smoke-free campus at Qatar University, 2022.

Statements	Students (n = 306)					Staff (n = 107)				
	Strongly Support	Support	Neutral	Do not support	Strongly do not support	Strongly Support	Support	Neutral	Do not support	Strongly do not support
***Attitudes toward the implementation of a tobacco-free policy***										
No one, including faculty, staff, students, visitors, and contractors, should be allowed to smoke anywhere on campus	170 (55.6)	36 (11.8)	28 (9.2)	32 (10.5)	40 (13.1)	61 (57.0)	19 (17.8)	6 (5.6)	6 (5.6)	15 (14.0)
Tobacco products should be strictly prohibited only in indoor spaces at Qatar University	178 (58.2)	34 (11.1)	24 (7.8)	25 (8.2)	45 (14.7)	61 (57.0)	8 (7.5)	9 (8.4)	5 (4.7)	24 (22.4)
Tobacco products should be allowed in outdoor spaces at Qatar University	52 (17.0)	43 (14. 1)	60 (19.6)	46 (15.0)	105 (34.3)	22 (20.6)	18 (16.8)	19 (17.8)	7 (0.5)	41 (38.3)
There should be no indoor designated smoking areas at Qatar University	189 (61.8)	21 (6.9)	37 (12.1)	25 (8.2)	34 (11.1)	77 (72.0)	7 (6.5)	8 (7.5)	9 (8.4)	6 (5.6)
There should be no outdoor designated smoking areas at Qatar University	115 (37.6)	39 (12.7)	47 (15.4)	55 (18.0)	50 (16.3)	46 (43.0)	11 (10.3)	16 (15.0)	14 (13.1)	20 (18.7)
The policy should include any tobacco product (such as e-cigarettes and chewed tobacco), not just cigarettes	186 (60.8)	39 (12.7)	30 (9.8)	22 (7.2)	29 (9.5)	65 (60.7)	14 (13.1)	15 (14.0)	6 (5.6)	7 (6.5)
The policy should cover cigarettes only	24 (7.8)	20 (6.5)	49 (16.0)	58 (19.0)	155 (50.7)	15 (14.0)	10 (9.3)	19 (17.8)	12 (11.2)	51 (47.7)
The university’s tobacco policy should be broadcasted to the entire QU community	215 (70.3)	33 (10.8)	41 (13.4)	7 (2.3)	10 (3.3)	79 (73.8)	13 (12.1)	13 (12.1)	0 (0.0)	2 (1.9)
The tobacco policy should be incorporated in the faculty and staff contracts	180 (58.8)	39 (12.7)	45 (14.7)	20 (6.5)	22 (7.2)	49 (45.8)	14 (13.1)	17 (15.9)	9 (8.4)	18 (16.8)
The tobacco policy should be incorporated in student orientation	194 (63.4)	47 (15.4)	40 (13.1)	9 (2.9)	16 (5.2)	64 (59.8)	21 (19.6)	14 (13.1)	1 (0.9)	7 (6.5)
The Tobacco -Free Policy should be incorporated in the Student Handbooks	180 (58.8)	59 (19.3)	34 (11.1)	14 (4.6)	19 (6.2)	69 (64.5)	19 (17.8)	10 (9.3.)	1 (0.9)	8 (7.5)
The Tobacco -Free Policy should be incorporated in QU contractor contracts and induction programs for new contractor employees	171 (55.9)	54 (17.6)	46 (15.0)	18 (5.9)	17 (5.6)	58 (54.2)	21 (19.6)	14 (13.1)	3 (2.8)	11 (10.3)
I support clear penalties for persons violating the tobacco policy	168 (54.9)	47 (15.4)	46 (15.0)	15 (4.9)	30 (9.8)	63 (58.9)	20 (18.7)	10 (9.3)	5 (4.7)	9 (8.4)
Penalties should be introduced, slowly, into the policy implementation to ensure that QU community complies with the policy	167 (54.6)	58 (19.0)	37 (12.1)	15 (4.9)	29 (9.5)	59 (55.1)	19 (17.8)	12 (11.2)	9 (8.4)	8 (7.5)

***Attitudes towards support for a 100 % tobacco-free and smoke-free campus***										
To what extent do you support your campus becoming 100 % smoke-free, tobacco-free, and vape-free, with all tobacco product use prohibited on campus?	196 (64.1)	35 (11.4)	33 (10.8)	12 (3.9)	30 (9.8)	70 (65.4)	12 (11.2)	8 (7.5)	7 (6.5)	10 (9.3)

Among students, the lowest mean score (24.1) indicating a negative attitude towards the implementation of the policy was reported among those using both traditional and electronic cigarettes (Table 3). Among faculty, the lowest score (24.5) was reported among users of electronic cigarettes. A number of sociodemographic factors were related to attitude scores towards implementation of a tobacco free policy. Among students, a lower score was significantly associated with being male (p = 0.041) and with being a current user of any type of tobacco in multivariable regression. Married students showed a more positive attitude towards the implementation of a tobacco- free policy compared to unmarried students (p < 0.001). Students having one family member who smokes and those having at least one friend who smokes had significantly lower scores when measuring the crude associations (p = 0.003 and p < 0.001, respectively), but not in adjusted analysis. Among faculty/staff, Qatari nationals showed more negative attitudes towards the policy than non-Qataris in the adjusted association (p = 0.022), and tobacco users had significantly lower scores compared to non-smokers when measuring both the crude and the adjusted associations (p < 0.05). Having a close friend who smokes was significantly associated with lower support for the policy in bivariate but not multivariate analysis.

**Table 3:** Crude and adjusted associations between predictor variables and overall score for attitudes toward the implementation of a tobacco-free policy at Qatar University, 2022.

Characteristic	Students (n = 306)							Staff (n = 107)						
	Crude				Adjusted			Crude				Adjusted		
	Mean	B (95 % CI)	SE	P-value	B (95 % CI)	SE	P-value	Mean	B (95 % CI)	SE	P-value	B (95 % CI)	SE	P-value
**Age (years) – students**														
18–19	40.9	Ref			Ref									
20–21	41.3	0.4 (−3.1, 3.9)	1.8	0.835	1.3 (−1.5, 4.0)	1.4	0.371							
22–24	36.9	−4.0 (−7.9, −0.1)	2	0.047	−0.5 (−3.8, 2.7)	1.6	0.743							
>24	39.4	−1.5 (−5.4, 2.4)	2	0.454	−0.8 (−4.8, 3.3)	2	0.71							
**Age (years) – staff**														
<34								37.8	Ref			Ref		
34–40								43.8	6.0 (0.3, 11.8)	2.9	0.041	5.2 (−0.3, 10.8)	2.8	0.066
41–48								36.5	−1.3 (−6.9, 4.4)	2.9	0.661	2.2 (−3.2, 8.0)	2.9	0.397
>48								43.4	5.7 (−0.3, 11.6)	3	0.063	4.2 (−1.9, 10.3)	3.1	0.181
**Gender**														
Female	42.1	Ref			Ref			42.3	Ref			Ref		
Male	36.4	−5.7 (−8.4, −3.0)	1.4	<0.001	−2.7 (−5.3, −0.1)	1.3	0.041	37	−5.3 (−9.6, −1.0)	2.2	0.016	−6.0 (−10.1, −1.8)	2.1	0.005
**Education level**														
Undergraduate	40	Ref			Ref			39.9	Ref			Ref		
Graduate – below PhD	40.1	0.1 (−5.2, 5.4)	2.7	0.97	−1.3 (−6.0, 3.3)	2.4	0.576	41.5	1.7 (−4.3, 7.7)	3.1	0.583	−2.5 (−8.0, 3.1)	2.8	0.382
Graduate – PhD	36.3	−1.0 (−5.5, 3.5)	2.3	0.663	−0.5 (−7.6, 6.6)	3.6	0.888	38.7	−1.1 (−7.2, 5.0)	3.1	0.72	−5.5 (−11.6, 0.6)	3.1	0.077
**Nationality**														
Non-Qatari	41.2	Ref			Ref			41.2	Ref			Ref		
Qatari	38.3	−2.9 (−5.6, −0.2)	1.4	0.037	−1.9 (−4.7, 0.8)	1.4	0.174	36.2	−5.0 (−10.2, 0.2)	2.7	0.061	−6.4 (−11.8, −1.0)	2.8	0.022
**Marital status**														
Single	39.4	Ref			Ref			38	Ref			Ref		
Married	43.6	4.2 (0.1, 8.3)	2.1	0.048	6.6 (2.7, 10.5)	2	<0.001	41	3.0 (−2.4, 8.5)	2.8	0.276	0.7 (−4.7, 6.1)	2.8	0.793
Separated/divorced	*							34.4	−3.6 (−14.7, 7.5)	5.6	0.524	−2.3 (−12.3, 7.7)	5.1	0.651
**Monthly household income (QAR)**														
<10,000	41.6	3.7 (−0.4, 7.9)	2.1	0.079	1.3 (−2.2, 4.8)	1.8	0.46	39.5	0.8 (−11.9, 12.5)	6	0.899	4.7 (−6.0, 15.4)	5.5	0.386
10,000–19,999	41	3.5 (0.1, 6.8)	1.7	0.042	1.8 (−1.4, 5.0)	1.6	0.276	41	−0.4 (−12.3, 11.6)	6.1	0.953	4.4 (−6.3, 15.1)	5.5	0.424
20,000–30,000	41.3	4.0 (0.3, 7.8)	1.9	0.036	2.7 (−0.8, 6.1)	1.8	0.127	39.1	1.5 (−10.5, 13.6)	6.1	0.802	4.5 (−5.6, 14.7)	5.1	0.38
>30,000	37.6	Ref			Ref			40.3	Ref			Ref		
**College – students**														
Health Cluster	39.9	Ref			Ref									
Other colleges	40	0.1 (−2.7, 2.8)	1.4	0.971	0.1 (−2.1, 2.3)	1.1	0.91							
**At least one family member is a smoker**														
No	41.5	Ref			Ref			41.7	Ref			Ref		
Yes	37.3	−4.3 (−7.0, −1.5)	1.4	0.003	−1.8 (−4.1, 0.6)	1.2	0.149	38.4	−3.3 (−7.6, 1.0)	2.2	0.137	−1.0 (−5.1, 3.1)	2.1	0.639
**At least one close friend is a smoker**														
No	43.3	Ref			Ref			42.7	Ref			Ref		
Yes	35.3	−8.0 (−10.6, −5.4)	1.3	<0.001	−0.8 (−3.6, 1.9)	1.4	0.543	36.9	−5.8 (−10.0, −1.5)	2.2	0.008	0.1 (−4.3, 4.4)	2.2	0.975
**Currently used tobacco products status**														
None	44	Ref			Ref			43.6	Ref			Ref		
Traditional cigarettes only	26.3	−17.6 (−21.2, −14.0)	1.8	<0.001	−15.4 (−19.3, −11.5)	2	<0.001	29.5	−14.1 (−20.0, −8.2)	3	<0.001	−10.6 (−16.8, −4.4)	3.2	<0.001
Electronic cigarettes only	30	−14.0 (−21.3, −6.7)	3.7	<0.001	−12.0 (−19.3, −4.7)	3.7	0.001	24.5	−19.1 (–32.8, −5.4)	7	0.006	−20.2 (–33.9, −6.5)	7	0.004
Traditional and electronic cigarettes	24.1	−19.8 (−24.0, −15.7)	2.1	<0.001	−17.9 (–22.3, −13.4)	2.3	<0.001	28.8	−14.8 (–23.6, −6.0)	4.5	<0.001	−12.0 (−21.1, −3.0)	4.6	0.009
Other tobacco products	35	−9.0 (−14.4, −3.6)	2.8	<0.001	−7.6 (−13.1, −2.1)	2.8	0.007	32.3	−11.4 (−18.4, −4.3)	3.6	0.002	−14.9 (–22.1, −7.6)	3.7	<0.001

B = regression coefficient (slope); SE = standard error; CI = confidence interval; Ref = reference category; QAR = Qatari Riyal. Only one student indicated his/her marital status as divorced/separated, and the student was grouped under the “single” marital status group.

## 4 Discussion

The current study assessed the support levels for tobacco-free policies among faculty and students at Qatar University. The results of this study echo some findings from previous literature (Farran et al., 2021), as both found that faculty and students supported tobacco free campuses and the extent of support was higher for non-smokers relative to smokers. However, the current study reveals that the percentage of support from smokers is substantially lower in comparison to those reported in previous studies (Farran et al., 2021). The low percentage of support from smokers in this study may suggest that personal liberty is a strong norm amongst smokers in Qatar. Smoking liberties are a persistent issue when applying tobacco control policies and they often serve as barriers to public health policy (Cardador et al., 1995). Therefore, a deeper understanding of the reasons that explain the strong opposition of smokers towards tobacco free policies at Qatar University is warranted to facilitate future policy implementation.

In this study, the majority of participants, regardless of their smoking status, supported a partial implementation of the policy, where using tobacco products would be prohibited in indoor spaces while allowed outdoors. Participants in this study also supported the gradual implementation of penalties in order to encourage compliance. Evidence from other universities suggests that gradually changing implementation from partial to full could increases the support for a 100 % smoke-free and tobacco-free policy eventually. Examples of changed attitudes following the implementation of tobacco free policies include the American University in Beirut where support for partial policy implementation sharply declined among smokers and non-smokers (Farran et al., 2021). Another example is the increased support for bans on smoking in a U.S. university (Lechner et al., 2012). Therefore, we expect that the implementation of a 100 % tobacco free policy at Qatar University is likely to increase the positive attitudes towards the policy, decrease current support for only partial implementation, while also achieving the goals of reduced tobacco use and reduced littering (Seo et al., 2011, Lee et al., 2013).

This study contributes three novel findings that identify the differences in positive and negative attitudes towards tobacco free policies by gender, residence, and type of tobacco product used for tobacco users. The first novel finding is the higher support for tobacco free policies among females relative to males. There are two explanations for this finding. First, the prevalence of smoking is lower among females in comparison to males (WHO, 2018), and non-smokers tend to support tobacco free policies more than smokers (Bartington et al., 2020, Forden and Carrillo, 2016). Second, females are more persistent with quit attempts, and their support for a tobacco free policy is logical as it constitutes a positive health policy action to facilitate smoking abstinence (Chinwong et al., 2018). This has implications for implementing tobacco free policies as it suggests that focusing on increasing awareness about the importance of policies among males may increase positive attitudes towards such policies. However, further studies are needed to confirm whether females consistently support tobacco free policies more than males.

A second novel finding is the fact that non-Qataris were found to have a higher positive attitude towards tobacco free policies compared to Qataris. This suggests that increased awareness about the importance of tobacco free policies among Qataris may increase support for such policies. Social marketing campaigns are effective in changing attitudes about smoking (Hefler et al., 2020). Such campaigns have the potential to bridge the gap in support for tobacco free policies between Qataris and non-Qataris, especially if focused on portraying tobacco free campuses as a positive reputational gain for the nation.

A third important note is the consistently negative attitudes towards tobacco free policies by tobacco users irrespective of the types of products that they use, especially, smokeless tobacco users where 100 % of users had negative attitudes about tobacco free policies. The disproportionately high negative attitude towards tobacco free policies by smokeless tobacco users has two potential explanations. Because it is not combustible and its use is arguably harmless or at least less harmful to the health of non-users, policies against smokeless tobacco use may have been perceived as excessive. The other explanation may be grounded in the idea that smokeless tobacco is considered a harm-reduction product when compared to cigarette smoking (Berg et al., 2015). Future research may be needed to better understand the factors that form the negative attitude of smokeless tobacco users about tobacco free policies. Nevertheless, including smokeless tobacco within the definition of tobacco free policies may be justified as a way to minimize exposure to the sight of smokeless tobacco use and de-normalizing tobacco use in general.

A noteworthy but not surprising finding is that students reported higher rates of novel tobacco products use such as e-cigarettes, HeatSticks, and nicotine pouches compared to staff. The appeal and popularity of novel tobacco products have been increasing among young adults when compared to traditional combustible cigarettes. Multiple studies demonstrated that youth and young adults tend to experiment with other tobacco products. Similar to our study, the use of electronic cigarettes is higher among youth and young adults when compared to older age groups (Villanti et al., 2017). Furthermore, the use of flavored tobacco products specifically, was found to be highest in youth (80 %) and young adult users (73 %) when compare to older adults ≥ 65 years (29 %) in a cross-sectional analysis of data from 45,971 adults and youth ≥ 12 years. Beside e-cigarettes and combustible cigarettes, youth also tend to experiment with products like waterpipe and cigars (Barrington-Trimis et al., 2016).

The current study has three limitations. First, it was conducted at one university in the Middle East, and the findings may not be generalizable to universities in settings that are substantially different. Further studies in other Middle Eastern countries are needed. Second, the study is cross sectional and limits conclusions with respect to changed attitudes over time. Conducting longitudinal studies, especially ones that look at attitudes before and after implementing tobacco free policies can provide stronger conclusions in support of tobacco free policies. Third, although the study highlights useful information on attitudes towards tobacco free policies, it does not provide insight into the beliefs that underlie each set of attitudes. Beliefs are specific and targeting them is effective for the purposes of understanding barriers toward tobacco free policies in future studies (Perloff, 2021).

## 5 Conclusion

The implementation of 100 % tobacco-free policies is a vital step towards promoting a healthier campus environment. Promoting such a policy requires a detailed understanding of the attitudes towards its individual components in order to tailor messaging and promotion interventions based on the issues relevant to the various stakeholders on a campus.

## Credit authorship contribution statement

**Ghadir Fakhri Al-Jayyousi:** Conceptualization, Funding acquisition, Investigation, Methodology, Project administration, Writing – original draft. **Mujahed Shraim:** Conceptualization, Formal analysis, Investigation, Methodology. **Diana Alsayed Hassan:** Conceptualization, Methodology, Writing – original draft, Writing – review & editing. **Mohammed Al-Hamdani:** Writing – original draft. **Rana Kurdi:** Writing – original draft. **Noor Ahmed Hamad:** Formal analysis, Writing – original draft. **Hanan F. Abdul-Rahim:** Conceptualization, Funding acquisition, Methodology, Supervision, Writing – review & editing.

## Declaration of competing interest

The authors declare that they have no known competing financial interests or personal relationships that could have appeared to influence the work reported in this paper.

## Data Availability

Data will be made available on request.

## Footnotes

1 Institutional Research and Effectiveness Department, Strategy and Development Office. Fall 2023 Semester Analysis (available on QU SharePoint). October 2023.
